# Measurements on radiation shielding efficacy of Polyethylene and Kevlar in the ISS (Columbus)

**DOI:** 10.1093/jrr/rrt198

**Published:** 2014-03

**Authors:** L. Di Fino, M. Larosa, V. Zaconte, M. Casolino, P. Picozza, L. Narici

**Affiliations:** Department of Physics, University of Rome Tor Vergata and INFN Tor Vergata, Rome, Italy

**Keywords:** space radiation, heavy ions, shielding, polyethylene, kevlar

## Abstract

The study and optimization of material effectiveness as radiation shield is a mandatory step toward human space exploration. Passive radiation shielding is one of the most important element in the entire radiation countermeasures package.

Crewmembers will never experience direct exposure to space radiation; they will be either inside some shelter (the spacecraft, a ‘base’) or in an EVA (Extra Vehicular Activity) suit. Understanding the radiation shielding features of materials is therefore an important step toward an optimization of shelters and suits construction in the quest for an integrated solution for radiation countermeasures.

Materials are usually tested for their radiation shielding effectiveness first with Monte Carlo simulations, then on ground, using particle accelerators and a number of specific ions known to be abundant in space, and finally in space.

Highly hydrogenated materials perform best as radiation shields. Polyethylene is right now seen as the material that merges a high level of hydrogenation, an easiness of handling and machining as well as an affordable cost, and it is often referred as a sort of ‘standard’ to which compare other materials' effectiveness.

Kevlar has recently shown very interesting radiation shielding properties, and it is also known to have important characteristics toward debris shielding, and can be used, for example, in space suits.

We have measured in the ISS the effectiveness of polyethylene and kevlar using three detectors of the ALTEA system [ 1– 3] from 8 June 2012 to 13 November 2012, in Express Rack 3 in Columbus. These active detectors are able to provide the radiation quality parameters in any orbital region; being identical, they are also suitable to be used in parallel (one for the unshielded baseline, two measuring radiation with two different amounts of the same material: 5 and 10 g/cm^2^).

A strong similarity of the shielding behavior between polyethylene and kevlar is documented. We measured shielding providing as much as ∼40% reduction for high Z ions. In Fig. 1, the integrated behavior (3 ≤LET ≤ 350 keV/µm) is shown (ratios with the baseline measurements with no shield) both for polyethylene and kevlar, in flux, dose and dose equivalent.

The measured reductions in dose for the 10 g/cm^2^ shields for high LET (>50 keV/µm, not shown in the figure) are in agreement with what found in accelerator measurements (Fe, 1 GeV) [4]. The thinner shielding (5 g/cm^2^) in our measurements performs ∼2% better (in unit areal density).

**Fig. 1.**
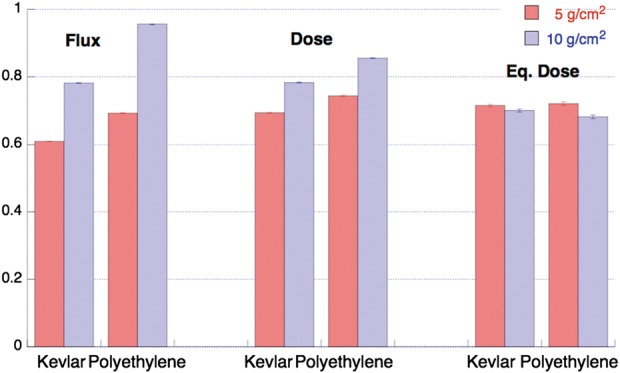
Integrated behavior (3 ≤ LET ≤ 350 keV/μm) of Flux, Dose and Equivalent Dose. The ratios with the baseline measurements with no shield are shown, both for Kevlar and Polyethylene as measured with the two different material thicknesses.

Integrated behavior (3 ≤ LET ≤ 350 keV/μm) of Flux, Dose and Equivalent Dose. The ratios with the baseline measurements with no shield are shown, both for Kevlar and Polyethylene as measured with the two different material thicknesses.
